# Preoperative Hyperlactatemia Predicts Mortality in Acute Stanford Type A Dissection: A 16-Year-Period, Single-Center, Retrospective Study

**DOI:** 10.3390/jcm14113619

**Published:** 2025-05-22

**Authors:** Nazan Puluca, Christian König, Gunther Wiesner, Birgit Waschulzik, Keti Vitanova, Markus Krane, Johannes Böhm

**Affiliations:** 1 Department of Cardiovascular Surgery, German Heart Center Munich, Technical University of Munich, 80636 Munich, Germany; puluca@dhm.mhn.de (N.P.); koenig@dhm.mhn.de (C.K.); vitanova@dhm.mhn.de (K.V.); krane@dhm.mhn.de (M.K.); 2 Insure (Institute for Translational Cardiac Surgery), Department of Cardiovascular Surgery, German Heart Center Munich, Technical University of Munich, 80333 Munich, Germany; 3 Institute of Cardioanesthesiology, German Heart Center Munich, Technical University of Munich, 80333 Munich, Germany; wiesner@dhm.mhn.de; 4 Institute for AI and Informatics in Medicine, Technical University of Munich School of Medicine, 81675 Munich, Germany; birgit.waschulzik@tum.de; 5 DZHK (German Center for Cardiovascular Research), Partner Site Munich Heart Alliance, 80636 Munich, Germany

**Keywords:** lactate, ischemia, dissection, malperfusion, cardiac surgery

## Abstract

**Background:** Acute Stanford Type A aortic dissection (ATAAD) is a devastating disease requiring immediate surgery. A life-threatening complication hereby represents organ malperfusion. Lactate is a product of anaerobic glycolysis indicating organ malperfusion. The current study analyzes preoperative lactate acidosis as a surrogate marker for patients’ outcome after surgery for ATAAD over a 15-year period. **Methods:** In a single-center setting, 306 consecutive patients, who underwent surgery for ATAAD between 2000 and 2016, were analyzed retrospectively. Serum lactate measurements were taken before surgery. To define a simple cut-point of the predictor lactate, the maximally selected rank statistics method was used. **Results:** Median survival was 9.3 ± 0.5 and CI 95% [8.3–10.2] years. Mean lactate levels were 1.95 mmol/L ± 2.19 mmol/L (range: 0.15–19.27 mmol/L). Patients with a lactate level > 3.71 mmol/L had a higher 30-day mortality compared to patients with lactate levels ≤ 3.71 mmol/L (51.5% versus 18.7%). In a logistic regression model adjusted for clinical baseline characteristics at index procedure, lactate levels > 3.71 mmol/L reached the highest Odd for 30-day mortality of all tested risk factors (OR = 7.292; CI95% [3.029–17.555]; *p* < 0.0001). Analyzing the overall mortality, the early effect of lactate level > 3.71 mmol/L persists. The HRs for overall mortality, however, revealed substantially lower effects (HR = 2.772; (CI95% [1.689–4.550]; *p* < 0.0001). In patients who survived the first 30 days postoperatively, no clinical parameter other than age had a significant impact on survival, including lactate > 3.71 mmol/L (*p* = 0.494). **Conclusions:** In patients with ATAAD, preoperative lactate represents an easily obtainable surrogate marker for organ malperfusion. A preoperative lactate level > 3.71 mmol/L depicts the strongest marker for early mortality after surgery.

## 1. Introduction

Acute Stanford Type A aortic dissection (ATAAD) represents a critical and often catastrophic cardiovascular emergency, characterized by a high risk of mortality. Without prompt surgical intervention, the condition has a mortality rate of approximately 22% within the first 24 h of symptom onset, escalating to nearly 100% in the subsequent days if left untreated [1]. This underscores the urgency of immediate diagnosis and management.

Despite advances in surgical techniques and perioperative care, the 30-day postoperative mortality for patients undergoing surgery for ATAAD remains significant, ranging from 8% to 30%. This variability is influenced by several clinical factors, including the extent and location of the dissection, the presence of pre-existing comorbidities, delays between diagnosis and surgical intervention, and the development of malperfusion syndromes and subsequent organ failure [2]. Ongoing research efforts have focused on identifying and analyzing individual risk factors, with the goal of creating more precise and reliable risk stratification tools for clinical use.

Among the most severe and life-threatening complications associated with ATAAD is organ malperfusion, which dramatically worsens the patient’s prognosis [3,4,5]. Visceral malperfusion is observed in approximately 16% to 33% of ATAAD cases [6], while lower limb ischemia occurs in around 15% of patients [7]. Furthermore, acute kidney injury (AKI), another common and serious manifestation of malperfusion, is reported in 20% to 67% of cases [8].

Lactate, a byproduct of anaerobic glycolysis, has long been recognized as a biochemical marker of tissue hypoperfusion and ischemia [9]. Elevated serum lactate levels reflect a shift to anaerobic metabolism, typically triggered by inadequate oxygen delivery to tissues. While the use of lactate as a biomarker for ischemia has been proposed in previous studies, comprehensive data regarding its prognostic value—particularly in relation to long-term outcomes and mortality in ATAAD patients—remain limited [6,10].

The present study aims to address this gap by evaluating the utility of serum lactate levels as a surrogate marker for organ malperfusion in ATAAD. Specifically, it seeks to determine whether a defined lactate threshold can be identified that correlates with increased 30-day mortality and impacts long-term survival, thereby offering clinicians a potential tool for early risk stratification and improved patient management.

## 2. Methods

The study protocol was approved by the local Ethics Committee of the Medical School of the Technical University of Munich (2022-400-S-NP), ensuring that the research adhered to the highest ethical standards for patient care and confidentiality. We included all consecutive patients who underwent surgery for ATAAD at the German Heart Center in Munich using cardiopulmonary bypass between 2000 and 2016. Data were obtained from an ongoing quality assessment program. All medical records, including operative reports and in-hospital and outpatient clinical notes, were reviewed.

In total, all patients who underwent surgery (*n* = 323) for Acute Stanford Type A Aortic Dissection (ATAAD) between the years 2000 and 2016 were included in this study. These patients were selected based on the availability of complete clinical data and their surgical management at our institution. However, 17 patients were excluded from the analysis due to the absence of preoperative lactate measurements, ensuring that only those with complete data (*n* = 306) were considered for the final analysis.

Serum lactate levels were measured 30–60 min prior to skin incision, providing a baseline marker for ischemic status at the time of surgical intervention.

All patients included in this study underwent surgery for ATAAD with cardiopulmonary bypass (CPB) according to our institutional standards, which follow established guidelines for the management of ATAAD in terms of surgical technique, patient monitoring, and post-operative care.

## 3. Statistical Analysis

Continuous data were described by mean ± standard deviation (SD) and categorical data by absolute and relative frequencies. Survival curves for overall mortality and 30-day mortality were plotted using the Kaplan–Meier method and compared by log rank test, as appropriate.

The ‘coin’ package of the R program version 4.1.2 was used for the evaluation of the cut-off point and Kaplan–Meier analyses [11]. To determine two groups of observations with respect to a simple cut-point of the predictor lactate, the maximally selected rank statistics method from the maxstat R package was used [12,13]. For statistical analysis, NCSS 20 statistical software and IBM SPSS 28 statistical software were used.

Cox regression analyses for overall mortality and multiple logistic regression analysis for 30-day mortality were applied. Potential candidate factors were chosen particularly regarding their clinical relevance as age and gender and parameters, which represent the extent of surgery, e.g., cardiopulmonary bypass time (CPB), surgery on the aortic arch, and aortic stenting. All analyses were conducted two-sided, using a 5% level of significance, and 95% confidence intervals (CIs) were calculated for relevant effect sizes.

## 4. Results

**Figure 1:**
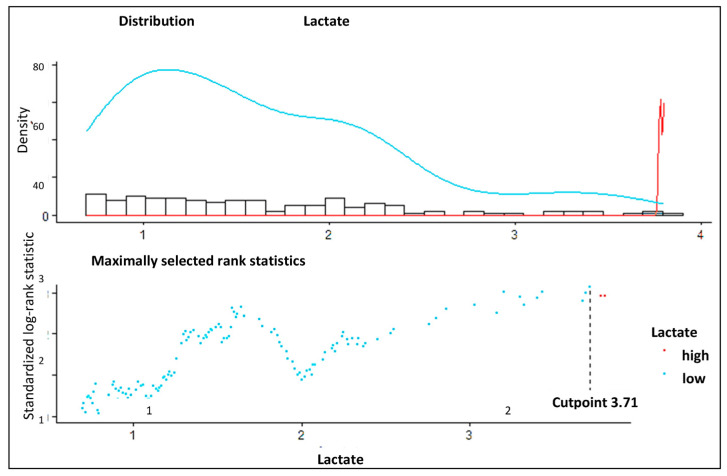
Determination of the cut-off point using maximally selected rank statistics. The optimum cut-off calculation of lactate level using the cut-off plot for lactate. The low levels are indicated in blue, and the high levels are indicated in red.

In total, 306 patients underwent surgery for ATAAD from 2000 to 2016 at our institution and met the inclusion criteria. Patients’ characteristics and surgical details are given in Table 1. Aortic valve replacement was performed in 111 patients (36.3%), and surgery of the aortic valve (SAVR or David procedure) was necessary in 131 (42.8%) patients. The mean cardiopulmonary bypass time was 195 ± 83 min. In 252 (82.4%) patients, circulatory arrest was performed with an average time of 38.0 ± 29.9 min (Table 1). Another aspect of special interest was the distinction of a low- and high-risk group regarding the level of lactate [mmol/L]. Maximally selected rank statistics revealed a cut-off point of 3.71 [mmol/L] (maxT = 3.1337, *p*-value = 0.028) (Figure 1).

## 5. Thirty-Day Mortality and Survival

**Figure 2:**
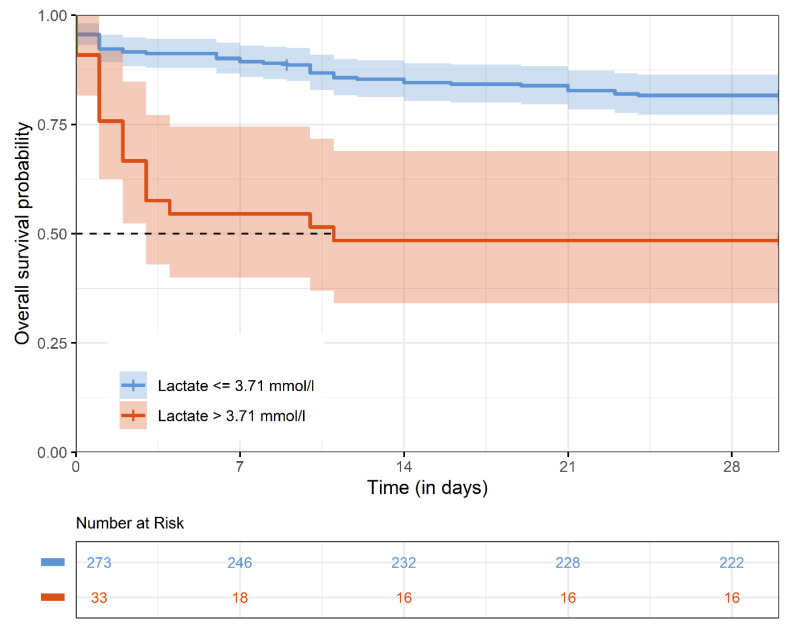
Kaplan–Meier estimates for 30-day survival. The Kaplan–Meier survival curve represents 30-day survival stratified by preoperative lactate levels (>3.71 mmol/L vs. ≤3.71 mmol/L).

The overall 30-day mortality was 21.4%. Patients with a lactate level > 3.71 mmol/L suffered from a much higher 30-day mortality rate compared to patients with lactate levels ≤ 3.71 mmol/L (51.5% versus 18.7%) (Figure 2).

**Figure 3:**
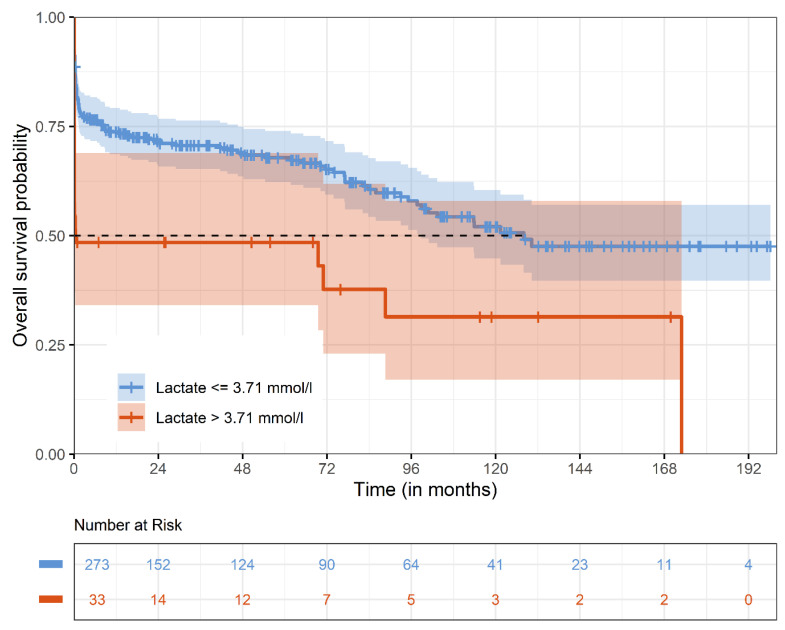
Kaplan–Meier estimates for survival. The Kaplan–Meier survival curve represents overall survival stratified by preoperative lactate levels (>3.71 mmol/L vs. ≤3.71 mmol/L).

Overall, median survival was 9.3 ± 0.5; CI 95% [8.3–10.2] years. Kaplan–Meier estimates for survival at 1 year, 5 years, and 10 years was 71.0% ± 0.03, 65.8% ± 0.03, and 49.8% ± 0.04, respectively. Kaplan–Meier estimates for survival stratified for lactate levels above and below 3.71 mmol/L are given for the whole observational period (Figure 3) and for the first 30 days after surgery (Figure 3). Survival in patients with lactate levels above 3.71 mmol/L at 1 year, 5 years, and 10 years were 48.5% ± 0.87, 48.5% ± 0.87 and 31.4% ± 0.98 compared to survival in patients with lactate levels below 3.71 mmol/L at 1 year, 5 years, and 10 years with 73.7% ± 0.27; 68.1% ± 0.3, and 52.8% ± 0.4 (*p* < 0.0001).

## 6. Risk Factor Analysis

Multiple logistic regression analyses for survival are presented for 30-day mortality (Table 2A,B) and overall mortality (Table 2A,B). For the 30-day period, lactate levels of >3.71 mmol/L exhibit the highest odds ratio (OR) of all tested risk factors (OR = 7.292; CI 95% [3.029–17.555]; *p* < 0.0001; Table 2A,B) and thereby depict the strongest predictor for 30 days mortality.

**Figure 4:**
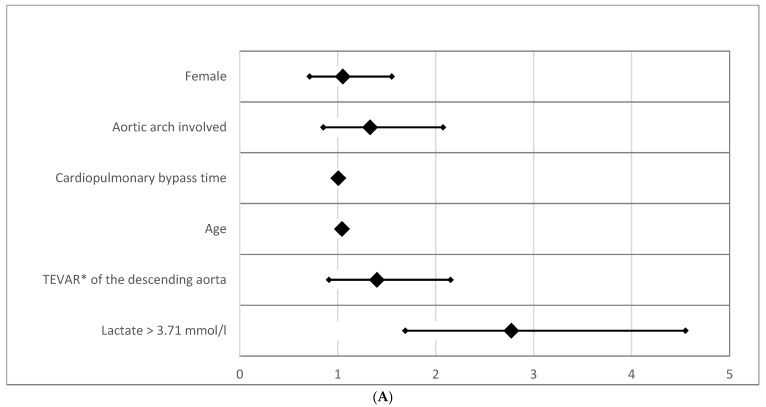

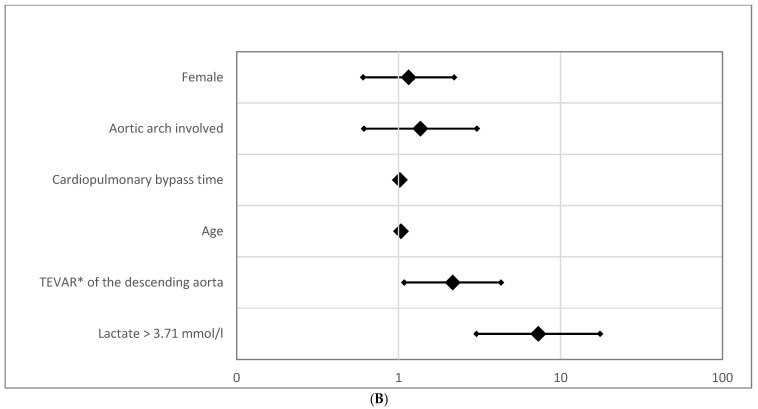
(**A**) Odds ratio for overall mortality. The forest plot displays odds ratios (ORs) for overall mortality based on preoperative lactate levels and other clinical parameters. (**B**) Odds ratio for 30-day mortality (logarithmic scaling). The forest plot represents odds ratios (ORs) for 30-day mortality in a logarithmic scale. Abbreviation: TEVAR*, Thoracic Endovascular Aortic Repair.

Analyzing the overall mortality, the early effect of lactate level > 3.71 mmol/L persists. The HRs for overall mortality, however, revealed substantially lower effects (HR = 2.772; (CI95% [1.689–4.550]; *p* < 0.0001). If only patients who survived the first 30 days postoperatively were included, no clinical parameter other than age had a significant impact on survival, including lactate > 3.71 mmol/L (*p* = 0.494). Forrest plots for overall mortality and 30-day mortality are given in Figure 4A,B.

## 7. Discussion

Surgical management of Acute Stanford Type A aortic dissection (ATAAD) continues to represent a significant challenge in both clinical practice and cardiovascular research due to the complexity, urgency, and high-risk nature of the condition [14]. Despite ongoing advances in surgical techniques, anesthetic management, and perioperative care, patient outcomes remain highly variable. This has led to the development of several risk stratification models aimed at identifying patients at increased risk of mortality and postoperative complications.

Among the most recognized scoring systems are the Penn Classification, the GOREISHI score, and the German Registry for Acute Aortic Dissection Type A (GERAADA) score. These tools incorporate a wide range of individual clinical variables—such as the presence of malperfusion, hemodynamic instability, age, comorbidities, and timing of intervention—to generate a composite risk profile that can help guide clinical decision-making and prognosis estimation [5,15].

However, despite their utility, these risk stratification scores have notable limitations in the context of real-time emergency care. Due to the inherent complexity and the number of data points required for accurate calculation, they may not be readily applicable in the acute setting, particularly in the critical preoperative window when time is of the essence.

Other diagnostic tools like advanced imaging techniques are necessary but are also elaborate and time-consuming.

Given these constraints, there is a growing interest in identifying simpler, more accessible biomarkers that can provide rapid prognostic information without requiring complex computations. One such candidate is serum lactate, a readily measurable laboratory parameter that reflects systemic hypoperfusion and anaerobic metabolism. The assessment of lactate acidosis may offer a cost-effective, rapid, and widely available tool that complements existing clinical evaluations.

In this context, evaluating the prognostic significance of elevated lactate levels in patients with ATAAD may provide critical insights into patient risk prior to surgery. By correlating serum lactate concentrations with early and long-term outcomes, clinicians may be better equipped to identify high-risk patients, optimize surgical timing, and improve resource allocation—even in high-pressure emergency environments where time and information are limited.

## 8. The Role of Organ Malperfusion

Hyperlactatemia, defined as elevated levels of lactate in the blood, reflects a shift toward anaerobic metabolism, typically induced by tissue hypoxia and ischemia [16,17]. In the setting of acute cardiovascular pathology, such as acute Stanford Type A aortic dissection (ATAAD), hyperlactatemia is of particular concern, as it may indicate the presence of significant end-organ malperfusion and systemic ischemia.

In a broader context of cardiac surgery, Govender et al. demonstrated that rising intraoperative lactate levels were independently associated with increased morbidity and mortality following cardiac procedures [18]. However, their study was not specific to ATAAD patients and was primarily focused on short-term postoperative outcomes, limiting its applicability to this highly specialized and critically ill patient population.

The extent of organ injury has been shown to directly influence circulating lactate levels, further reinforcing lactate’s role as a biomarker of systemic stress and hypoperfusion [19]. In the context of ATAAD, hyperlactatemia is commonly attributed to inadequate perfusion of vital organs due to the compromised aortic anatomy. Malperfusion syndromes—affecting visceral, renal, or peripheral circulation—result in localized ischemia and thus drive lactate production [20]. In our analysis, we did not focus on the specific type of organ malperfusion but instead aimed to identify a general prognostic marker for the entire cohort. Although laboratory screening tests—such as liver and renal function values—were obtained immediately before surgery, these parameters were not included as preoperative prognostic markers, as they tend to worsen over time and may not accurately reflect the patient’s initial status. Demers et al. provided further evidence of the prognostic significance of lactate levels in cardiac surgery by identifying a strong correlation between peak lactate concentrations exceeding 4 mmol/L during cardiopulmonary bypass and adverse postoperative outcomes, including increased morbidity and mortality [21]. Complementing these findings, Bennett et al. observed that elevated lactate levels were associated not only with higher in-hospital mortality but also with increased 1-year mortality rates following cardiac surgery [22]. Their study proposed a preoperative lactate cut-off value in the range of 6.0 to 6.9 mmol/L as a potential threshold for identifying high-risk patients. However, one limitation of this study was the absence of preoperative lactate data for all patients, which may have impacted the robustness of their conclusions. Nevertheless, in their analysis, lactate emerged as the sole significant predictor of poor postoperative outcomes, underlining its potential role as a marker of end-organ ischemia.

Importantly, elevated lactate levels may not only serve as a prognostic indicator but could also influence intraoperative decision-making. Patients exhibiting signs of ischemia, as evidenced by elevated lactate concentrations, may benefit more from expedited and less extensive surgical procedures. These strategies aim to minimize operative time and thereby reduce the metabolic stress of prolonged surgery in already compromised patients. However, it is essential to recognize that the extent of the surgical approach is determined by multiple factors, including the quality of the aortic tissue, intraoperative findings, and the experience and preference of the operating surgeon.

Given the growing body of evidence linking rising lactate levels with worse clinical outcomes, there is a compelling need to define a specific lactate threshold that reliably predicts mortality. Establishing such a cut-off point would enhance risk stratification and could provide clinicians with an easily measurable and cost-effective tool to guide urgent management decisions in ATAAD. The specificity of lactate levels at particular thresholds for predicting mortality could thus hold significant clinical relevance, aiding in both prognostication and surgical planning in this high-risk population.

## 9. Cut-Off Level and the Prognostic Impact of Lactate Levels on Survival

The current study identified a lactate threshold of >3.71 mmol/L as a significant predictor of 30-day mortality in patients undergoing surgery for acute Stanford Type A aortic dissection (ATAAD). Although only a small proportion of patients—33 individuals (10.7%)—presented with lactate levels exceeding this threshold, these patients demonstrated a substantially higher 30-day mortality rate compared to those with lower lactate levels. Specifically, the 30-day mortality in the high lactate group was 51.5%, compared to just 18.7% in the control group (*p* < 0.0001). This stark contrast underscores the potential clinical importance of lactate as a prognostic biomarker in this patient population.

Further analysis through multivariate regression, adjusting for common risk factors associated with ATAAD, revealed that lactate levels above 3.71 mmol/L were the strongest independent predictor of 30-day mortality (odds ratio [OR] = 7.292; 95% confidence interval [CI] [3.029–17.555]; *p* < 0.0001). This finding highlights the robustness of lactate as a prognostic tool and reinforces its potential to serve as an early marker for patients at highest risk of poor outcomes.

When evaluating the impact of lactate on long-term survival, it became apparent that the early mortality effect of elevated lactate levels (i.e., those > 3.71 mmol/L) persists, though with a reduced effect over time. The hazard ratio (HR) for overall mortality was calculated at 2.772 (95% CI [1.689–4.550]; *p* < 0.0001), indicating that elevated lactate levels continue to influence survival, though less strongly than in the short term. This suggests that while high lactate levels are a strong predictor of immediate postoperative mortality, their prognostic value diminishes when considering overall long-term survival (Table 2A,B).

Interestingly, when the analysis was restricted to patients who survived the first 30 days postoperatively, no parameter, including lactate levels > 3.71 mmol/L, had a statistically significant impact on survival beyond this initial period. In fact, age was the only factor that remained significantly associated with survival, indicating that the early-phase mortality effects of hyperlactatemia may not fully capture the long-term prognosis in ATAAD patients (*p* = 0.494). This finding suggests that once patients pass the critical 30-day postoperative window, their survival is more strongly influenced by factors such as age, while lactate levels no longer appear to be a decisive factor in long-term outcomes.

Despite the diminished influence of lactate on long-term survival, the early impact of elevated lactate levels on overall mortality is profound. For patients with lactate levels > 3.71 mmol/L, only 48.5% survived the first year after surgery. In contrast, those with lactate levels ≤ 3.71 mmol/L demonstrated a comparable survival rate only after 10 years, with a 52.8% survival rate ± 0.4% (after 10 years). These data suggest that while short-term survival is highly sensitive to lactate levels, long-term prognosis becomes more favorable for patients with lower lactate levels.

Further investigation into other common risk factors for mortality in cardiac surgery revealed interesting patterns. For instance, aortic arch dissection and female gender were associated with increased odds ratios (ORs) for mortality but did not reach statistical significance (Table 2A,B). This finding contrasts with prior studies, where aortic arch dissection was often identified as a significant predictor of adverse outcomes [23,24]. Moreover, aortic stenting of the descending aorta increased short-term mortality risk (OR = 2.158; 95% CI [1.085–4.291]; *p* = 0.028) but, interestingly, did not affect long-term survival (*p* = 0.985). These results suggest that while certain surgical interventions may contribute to short-term risks, their long-term impact on survival is less pronounced.

The lactate cut-off point of 3.71 mmol/L used in our study was determined using the maximally selected rank statistics method via the maxstat package in R. This statistical approach identifies the optimal threshold that best separates the survival distributions of two groups based on the predictor—in this case, preoperative lactate levels. While this method offers an objective, data-driven means of cut-off selection, it is based on maximizing the difference in outcome (mortality) and thus may not reflect physiological thresholds applicable to all patient populations. In particular, the appropriateness of this cut-off for patients of different age groups is uncertain. Age-related physiological differences, including baseline metabolic rates and lactate clearance, can affect lactate dynamics.

Age and cardiopulmonary bypass (CPB) time, which have been consistently recognized as important risk factors in the literature, also showed the expected influence on mortality outcomes. However, their effect was somewhat less pronounced in this study, with only modestly increased odds ratios (OR = 1.052 and OR = 1.006, respectively). This suggests that while both age and CPB time remain critical factors in patient management, their contribution to long-term outcomes in ATAAD surgery may be less specific compared to biomarkers like lactate. These findings emphasize the more generalized prognostic impact of age and CPB time, underscoring their role as contributing but not definitive predictors of mortality.

## 10. Conclusions

In patients with Acute Stanford Type A aortic dissection (ATAAD), preoperative lactate levels serve as an easily obtainable marker of organ malperfusion and ischemia. A lactate level > 3.71 mmol/L is a strong predictor of early mortality following surgery. As lactate is cost-effective, simple to measure, and readily available, it provides clinicians with a quick and reliable assessment of ischemic status. This can help guide decision-making, especially in the urgent setting of ATAAD to identify high-risk patients.

## 11. Limitations

Within our study, there are certain limitations. The retrospective character of the study has obvious limits but offers the results of real-life surgery over a 15-year period. Larger, multi-center studies are necessary to confirm the current results. ATAAD affects multiple organ systems with a variety of interactions, potentially influencing lactate levels. Therefore, the heterogeneity of organ malperfusion limits the specificity of our analysis. Nevertheless, a lactate cut-off value may provide one important component to assess patient risk before surgery. Notably, our data identified a lower lactate cut-off with a significant impact on patient outcomes, suggesting that even lower lactate levels may be crucial in predicting surgery-related risks, although further research and external validation are needed to verify this finding.

## Figures and Tables

**Table 1:** Patient characteristics and perioperative results.

*n* = 306	Mean ± SD or *n* (%)
**Patient’s characteristics**	
Age (years)	59.44 ± 14.25 (18.12–92.16)
Female gender (*n*)	95 (30.9%)
Marfan (*n*)	13 (4.2%)
Lactate preoperative (mmol/L)	1.95 ± 2.19 (0.15–19.27)
**Lactate > 3.71 mml/L**	33 (10.7%)
Creatinine preoperative (mg/dL)	1.13 ± 0.5 (0.39–5.68)
Elevated liver parameters (at admission; *n*)	109 (35.5%)
Body mass index (BMI) (kg/m^2^)	26.5 ± 4.0
Left ventricular ejection fraction (%)	62 ± 16.1
Previous cardiac surgery (*n*)	29 (9.4%)
**Surgical and intraoperative data**	
Surgery on the aortic arch (*n*)	210 (68.4%)
SAVR * (*n*)	111 (36.3%)
Bentall procedure (total; *n*)	105 (34.2%)
Bentall procedure, mechanical SAVR (*n*)	32 (10.5%)
David procedure (*n*)	13 (4.2%)
TEVAR ** of the descending aorta	84 (27.4%)
Concomitant CABG *** (*n*)	54 (17.6%)
CPB **** time (minutes)	195 ± 83
Circulatory arrest (minutes) (*n* = 252; 82.4%)	38.0 ± 29.9
ECMO ***** (*n*; postoperative)	7 (2.3%)

* Surgical aortic valve replacement; ** total endovascular aortic replacement; *** coronary artery bypass grafting; **** cardiopulmonary bypass time; elevated liver parameters defined as bilirubin > 1.2 mg/dL, alanine aminotransferase > 50 mmol/L, and aspartate aminotransferase > 40 mmol/L; ***** extracorporeal membrane oxygenation. Statistical analyses included *t*-tests or Mann–Whitney U tests for continuous variables and chi-square or Fisher’s exact tests for categorical variables; values are expressed as mean ± SD, median (IQR), or *n* (%).

**Table 2:** A: Multivariate cox regression analysis for overall mortality; B: multivariate logistic regression analysis for 30-day mortality.

**A**					
	**B**	**Hazard Ratio (HR)**	**Lower Level (95% CI)**	**Upper Level (95% CI)**	***p*-Value**
Lactate > 3.71 mmol/L	1.020	2.772	1.689	4.550	<0.0001
TEVAR * of the descending aorta	0.336	1.399	0.910	2.151	0.126
Age	0.042	1.043	1.026	1.059	<0.0001
Cardiopulmonary bypass time	0.006	1.006	1.004	1.008	<0.0001
Aortic arch surgery	0.285	1.330	0.853	2.074	0.208
Female gender	0.050	1.051	0.713	1.551	0.800
**B**					
	**B**	**Odds Ratio (OR)**	**Lower Level (95% CI)**	**Upper Level (95% CI)**	***p*-Value**
Lactate > 3.71 mmol/L	1.987	7.292	3.029	17.555	<0.0001
TEVAR * of the descending aorta	0.769	2.158	1.085	4.291	0.028
Age	0.035	1.036	1.010	1.062	0.006
Cardiopulmonary bypass time	0.009	1.009	1.005	1.013	<0.0001
Aortic arch involved	0.311	1.365	0.612	3.044	0.447
Female gender	0.172	1.187	0.620	2.275	0.605
Constant	−6.096	0.002			<0.0001

Multivariate cox regression was performed to assess hazard ratios for mortality. Values are represented as HR (95% CI). Abbreviations: HR, hazard ratio; CI, confidence interval. Multivariate logistic regression was performed to assess odds ratios for early mortality. Values are represented as HR (95% CI). Abbreviations: HR, hazard ratio; CI, confidence interval; * TEVAR, Thoracic Endovascular Aortic Repair.

## Data Availability

The original contributions presented in this study are included in the article. Further inquiries can be directed to the corresponding author(s).
